# A holistic high-throughput screening framework for biofuel feedstock assessment that characterises variations in soluble sugars and cell wall composition in *Sorghum bicolor*

**DOI:** 10.1186/1754-6834-6-186

**Published:** 2013-12-23

**Authors:** Antony P Martin, William M Palmer, Caitlin S Byrt, Robert T Furbank, Christopher PL Grof

**Affiliations:** 1 School of Environmental and Life Sciences, University of Newcastle, University Drive, Callaghan NSW 2308, Australia; 2 Australian Research Council Centre of Excellence in Plant Cell Walls, Waite Research Institute, University of Adelaide, Glen Osmond, SA 5064, Australia; 3 CSIRO Plant Industry, High Resolution Plant Phenomics Centre, GPO Box 1600, Canberra ACT 2601, Australia

**Keywords:** FTIR, High-throughput, PLS modelling, Cell wall, Soluble sugar, Digestibility, *Sorghum*, Biofuel

## Abstract

**Background:**

A major hindrance to the development of high yielding biofuel feedstocks is the ability to rapidly assess large populations for fermentable sugar yields. Whilst recent advances have outlined methods for the rapid assessment of biomass saccharification efficiency, none take into account the total biomass, or the soluble sugar fraction of the plant. Here we present a holistic high-throughput methodology for assessing sweet *Sorghum bicolor* feedstocks at 10 days post-anthesis for total fermentable sugar yields including stalk biomass, soluble sugar concentrations, and cell wall saccharification efficiency.

**Results:**

A mathematical method for assessing whole *S. bicolor* stalks using the fourth internode from the base of the plant proved to be an effective high-throughput strategy for assessing stalk biomass, soluble sugar concentrations, and cell wall composition and allowed calculation of total stalk fermentable sugars. A high-throughput method for measuring soluble sucrose, glucose, and fructose using partial least squares (PLS) modelling of juice Fourier transform infrared (FTIR) spectra was developed. The PLS prediction was shown to be highly accurate with each sugar attaining a coefficient of determination (*R*^*2*^) of 0.99 with a root mean squared error of prediction (RMSEP) of 11.93, 5.52, and 3.23 mM for sucrose, glucose, and fructose, respectively, which constitutes an error of <4% in each case. The sugar PLS model correlated well with gas chromatography–mass spectrometry (GC-MS) and brix measures. Similarly, a high-throughput method for predicting enzymatic cell wall digestibility using PLS modelling of FTIR spectra obtained from *S. bicolor* bagasse was developed. The PLS prediction was shown to be accurate with an *R*^*2*^ of 0.94 and RMSEP of 0.64 μg.mgDW^-1^.h^-1^.

**Conclusions:**

This methodology has been demonstrated as an efficient and effective way to screen large biofuel feedstock populations for biomass, soluble sugar concentrations, and cell wall digestibility simultaneously allowing a total fermentable yield calculation. It unifies and simplifies previous screening methodologies to produce a holistic assessment of biofuel feedstock potential.

## Background

The photosynthetic fixation of atmospheric CO_2_ to produce sugars by C_4_ plants is an efficient conversion of sunlight into stored chemical energy. In grasses a large proportion of these sugars are stored either as soluble sugars, plant cell wall polymers, or starch. Maximising sugar yields from these three fractions for a broad spectrum of uses including animal feedstock, human nutrition, and second generation biofuels is imperative for efficient utilisation of plant productivity in the face of a burgeoning global population.

This study focused on the soluble sugar and cell wall fractions as a biofuel feedstock. Considering these two fractions, there are four main contributors that maximise total fermentable sugar yields of biofuel feedstocks: juice volume (*V*), sugar concentration ([*S*]), biomass (*B*), and digestibility (*D*). These attributes can be formulated as:

$$V.\left[S\right]+B.D=\mathrm{total}\;\mathrm{fermentable}\;\mathrm{sugar}\;\mathrm{yield}$$

A European Union EPOBIO project [1], and a more recent report [2], stipulate that there is a need for a high-throughput method for assessing cell wall digestibility [1,2]. Since these reports, a number of high-throughput digestibility assays have been developed [3-5]. These methodologies present feasible high-throughput platforms for screening biomass, however, do not present strategies for high-throughput quantification of total plant biomass (*B*), or of the soluble sugar fraction (*V* and [*S*]). In addition, analysis requires automated robotics which is costly, and uses wet chemistry assays that are, again, costly, use hazardous chemicals, and even when automated, can be time consuming. This study focused on quantifying all of these attributes (*V*, [*S*], *B*, and *D*) simultaneously, whilst maximising efficiency and reducing cost.

The principal components of cell walls are cellulose, hemicellulose, and lignin. The composition and arrangement of these carbohydrates and phenolic polymers is very diverse across plant species, organs, tissues, and even cell types [6]. With a strong focus on renewable energy being derived from plant biomass, the highly efficient C_4_ plants and more specifically monocot grasses have garnered significant interest in recent times as potential biofuel feedstocks [7]. A promising saccharification approach for plant biomass requires reduction to a uniform size, pretreatment with weak acid or alkali, followed by enzymatic hydrolysis to release sugars for fermentation. The commelinoid group of monocotyledons which includes the grasses, hence principal biofuel feedstock targets such as sugarcane, *Sorghum,* and *Miscanthus*, possess cell walls containing ferulic acid and glucuronoarabinoxylan as the major non-cellulosic polysaccharide. Furthermore, the grasses differ from other commelinoids in possessing (1,3;1,4)-β-glucans [8]. Cell walls of non-commelinoid monocotyledons and most dicotyledonous plants do not contain ferulic acid and the non-cellulosic polysaccharides are pectins and xyloglucans [6,9]. These differences in cell wall composition and architecture as well as the bonds between the principal components strongly influence ‘digestibility’ of the plant biomass, whether this be undertaken in a biofuels processing context or by rumen/gut microorganisms.

Analysis techniques based upon infrared (IR) spectroscopy are proving to be highly effective tools for the detailed resolution of plant cell wall composition. IR spectroscopy is based on the absorption of IR light by specific quantized vibrational energy states of bonds between atoms and within molecules [10]. Mid-infrared (MIR) absorption spectra (4,000 to 400 cm^-1^) define vibrational modes directly, generating sharp peaks that are more readily interpreted than near-infrared (NIR; 13,000 to 4,000 cm^-1^) or far-infrared (FIR; 10 to 400 cm^-1^) spectra [10]. Fourier transform MIR (FT-MIR or FTIR) spectra have been used to characterise molecules for approximately 100 years and were first used in the analysis of plant cell walls more than 30 years ago [11]. More recently, when coupled with multivariate data analysis techniques, FTIR spectroscopy has proven to be a robust and accurate method for high-throughput screening of cell wall mutations in experimental plant tissues, such as the model species *Arabidopsis thaliana*[12,13] and *Zea mays* coleoptiles [14,15], where even slight variations in the molecular structure of cell walls were detected. Recently, FTIR spectra have been used as a predictor for enzymatic hydrolysis of pre-treated biomass [16] and similarly, in the food industry to rapidly quantify sucrose, glucose, and fructose in juice including mango, apple, and sugarcane [17-19] as well as other foodstuffs such as honey [20]. Similarly, NIR has been used for rapid prediction of soluble sugars in sugarcane [21] and of biomass composition in *Miscanthus* and *Sorghum*[22,23].

**Figure 1:**
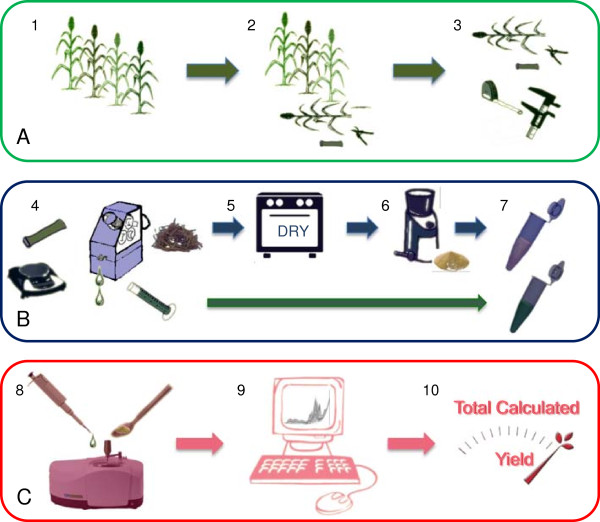
**High-throughput methodology flow chart. (A)** (1) Diverse plant population; (2) harvest the fourth internode; and (3) record in-field geometric measurements. **(B)** (4) Process into juice and bagasse; (5) dry bagasse; (6) grind dried tissue; and (7) process juice and bagasse. **(C)** (8) Collect FTIR spectra; (9) PLS prediction of FTIR spectra; and (10) calculate combined total fermentable sugar yield incorporating juice volume (*V*), sugar concentration ([*S*]), biomass (*B*), and digestibility (*D*) (see Additional file 1 for full protocol). FTIR, Fourier transform infrared; PLS, partial least squares.

Whilst these studies are suggestive of an application in high-throughput screening of biofuel feedstocks, there have been no reports incorporating this technology into a holistic, high-throughput assessment of biofuel feedstock potential. To this end, a screening strategy has been developed based upon stalk geometry, and partial least squares (PLS) predictive models of FT-MIR spectra collected from the soluble sugar and cell wall fractions. The methodology (Figure 1 and Additional file 1 for detailed protocol) was shown to be accurate for plants harvested at 10 days post-anthesis and enabled the identification of both high sugar accumulating and highly digestible lines leading to an assessment of total fermentable sugars in the model C_4_ monocot *Sorghum bicolor*. The use of FT-MIR for both soluble sugar concentration and cell wall digestibility prediction simplified the process and reduced set-up costs. Furthermore, the collection of FT-MIR, rather than NIR, spectra provides an opportunity for downstream spectral interpretation of cell wall modifications that lead to altered digestibility.

## Results

A detailed step-by-step protocol for undertaking the proposed screening method is available in Additional file 1. Briefly, the main stalk from each plant, harvested at approximately 10 days post-anthesis, was cut at the base of the first elongated above ground internode and laid along the length of a 4-meter ruler. Two heights and two diameters were recorded in a personal digital assistant (PDA) and the fourth internode from the base of the plant that had expanded more than 2 cm was sampled by cutting at the node with secateurs. Using this method, one person was able to sample approximately 100 internodes per hour. Samples were then pressed and the released juice was placed on the attenuated total reflectance (ATR) crystal of a portable FTIR spectrometer for spectral acquisition. The remaining bagasse was simultaneously placed in a drying oven followed by grinding, washing with water, and placing on the FTIR ATR crystal for spectral acquisition. Data was then batch processed by applying predictive models to FTIR spectra and incorporating data into yield calculations. An overview of this process is displayed in Figure 1 and the results describing the calibration and accuracy of each prediction process are presented below.

### High-throughput calculation of whole stalk biomass, sugar content, and cell wall hydrolysis yield

**Figure 2:**
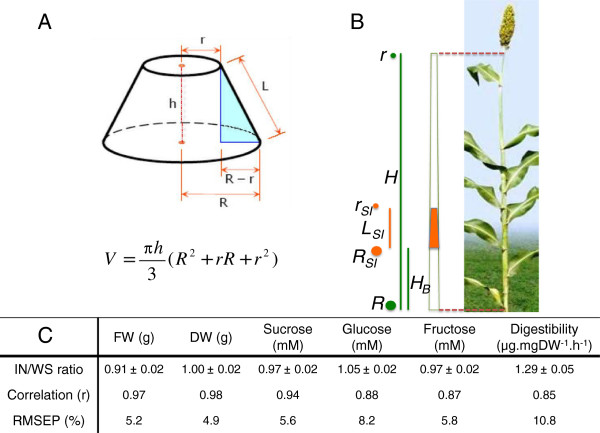
**Extrapolating sampled biomass characteristics to whole stalks. (A)***S. bicolor* stalks were assumed to approximate a conical frustum allowing a simple volumetric calculation. **(B)** The fourth internode from the base of the plant that had expanded more than 2 cm was harvested and the height (*H*), stalk radius at the base (*R*) and top of the stalk (*r*), the height to the bottom of the fourth internode (*H*_*B*_), and the length of the fourth internode (*L*) were recorded. Fresh weight (FW), dry weight (DW), sucrose, glucose, and fructose concentrations of pressed juice and digestibility of the lignocellulose were measured in the fourth internode at approximately 10 days post-anthesis. Using the volumetric ratio (*V*_*ratio*_), these measurements were extrapolated to whole stalks and compared to equivalent measures taken from the same whole stalk. **(C)** The ratio of extrapolated fourth internode measures to whole stalks (IN/WS ratio), their correlation (r), and the error in prediction (RMSEP, %) are tabulated. DW, dry weight; FW, fresh weight; RMSEP, root mean squared error of prediction.

For the purpose of calculating the whole stalk volume it was assumed that a *S. bicolor* stalk approximates a conical frustum (Figure 2). By simply measuring the radius at the top (*r*) and bottom (*R*) of the stalk and the stalk height (*H*), the volume of the whole stalk (*V*_*WS*_) could be derived using the equation for the volume of a conical frustum:

$$V_{\mathrm{WS}}=\frac{\mathrm{\pi H}}{3}\left(r^{2}+\mathrm{rR}+R^{2}\right)$$

Using only two additional measurements, length from the base of the stalk to the base of the sampled internode(s) (*H*_*B*_) and length of the sampled internode (*L*_*SI*_), the volume of the sampled internode (*V*_*SI*_) was calculated. Both measurements were acquired simply by using a ruler during stalk height measurement, whilst the base radius (*R*_*I*_) and top radius (*r*_*I*_) of the sampled internode(s) were derived using simple geometry to obtain the following equation:

$$V_{\mathrm{SI}}=\frac{\pi L_{\mathrm{SI}}}{3}\left(R_{I}^{2}+r_{I}R_{I}+r_{I}^{2}\right)$$

where $R_{I}=R-H_{B}\frac{R-r}{H}$ and $r_{I}=R-\left(H_{B}+L_{\mathrm{SI}}\right)\frac{R-r}{H}$

See Additional file 2 for full derivation.

The volumetric ratio of the sampled internode to the whole stalk was then calculated as:

$$V_{\mathrm{ratio}}=V_{\mathrm{SI}}/V_{\mathrm{WS}}$$

From this ratio, measurements taken on the sampled internode could be extrapolated to the whole stalk by simply dividing by *V*_*ratio*_. This, of course, is under the assumption that the concentration, or density of the measured variable in the sampled internode(s) is either equivalent to, or a fixed ratio of that in the whole stalk. In our case, the fourth internode from the base of the stalk was used as the sample internode since a stable relationship between sugar concentrations in the fourth internode and the whole stalk had been repeatedly observed in previous field measurements (unpublished data) and it has been shown that lower internodes in maize stalks within a genotype are less variable [24]. The relationship between fresh weight (FW), dry weight (DW), sugar concentration, and cell wall digestibility of the fourth internode and that of the whole stalk was determined using 15 mature, field grown sweet *S. bicolor* genotypes harvested at 10 days post-anthesis with a height and FW range of 237 to 338 cm and 228 to 941 g (Additional file 3), respectively (Figure 2). Four replicate, glasshouse grown ‘Rio’ sweet sorghum plants, also harvested at approximately 10 days post-anthesis, were used in the cell wall digestibility correlation calculations to supplement lost samples.

Calculations using *V*_*ratio*_ and measurements from the fourth internode were compared to those taken from the whole stalk. FW and DW returned values that were 91 ± 2% and 100 ± 2% of the whole stalk with correlations of 0.97 and 0.98, respectively. Sucrose, glucose, and fructose returned values that were 97 ± 2%, 105 ± 2%, and 97 ± 2% of the whole stalk with correlations of 0.94, 0.88, and 0.87, respectively. Digestibility as calculated from the fourth internode, however, gave values that were 129 ± 5% of the whole stalk with a correlation of 0.85 (Figure 2C). Whilst not all ratios approached 100%, all had small standard errors suggesting that these fixed ratios were consistent across genotypes, and could therefore be used in extrapolating measurements from the fourth internode to whole stalks.

Using these experimentally determined ratios, FW, DW, sucrose, glucose, fructose, and digestibility values were adjusted and the root mean squared error of prediction (RMSEP) was determined to be 5.2%, 4.9%, 5.6%, 8.2%, 5.8%, and 10.8%, respectively (Figure 2C). The calculation-based method proposed here is, therefore, accurate across the 15 examined genotypes and is suitable for assessing total biomass, juice volume, sugar content, and digestibility of whole stalks in a high-throughput screen of sweet *S. bicolor* populations. Whilst these correlations allow relatively accurate predictions to be made in a high-throughput manner, it should be noted that it is the nature of predictive modelling that cultivars or samples which do not follow the established rule, such as a lower stem-specific gene mutation, will not perform well in the model. Often these samples will be identified as outliers for more rigorous study; however, one must also accept that there is always a statistical probability that a phenotype of interest will not be detected.

To our knowledge, this is the first mathematical-based modelling strategy for assessing total stalk biomass in a high-throughput manner in *S. bicolor* or any other biofuel feedstock. Whilst high-throughput methods for assessing shoot biomass are being developed using two-dimensional images [25] this technology is not applicable to dense field plots with thick canopies and does not allow extrapolation of other measurements. The method presented here not only produced data on stalk geometry including height and stalk diameter which are traits of interest when breeding lines for increased biomass yields, but also greatly reduced processing time by eradicating the need to: strip whole stalks of their leaves and leaf sheaths; transport large stalks from the field to the laboratory; operate large balances; press juice from whole stalks; dry whole stalks in large drying ovens; and grind whole stalks in a large roller mill. In addition, since this approach relies on stalk geometry that resembles a conical frustum, it is suggested that it could be applied to other grasses with similar geometry such as sugarcane, maize, and millet; however, the fixed ratios would have to be separately determined in each species.

### FTIR prediction of sucrose, glucose, and fructose concentrations in stalk juice

**Figure 3:**
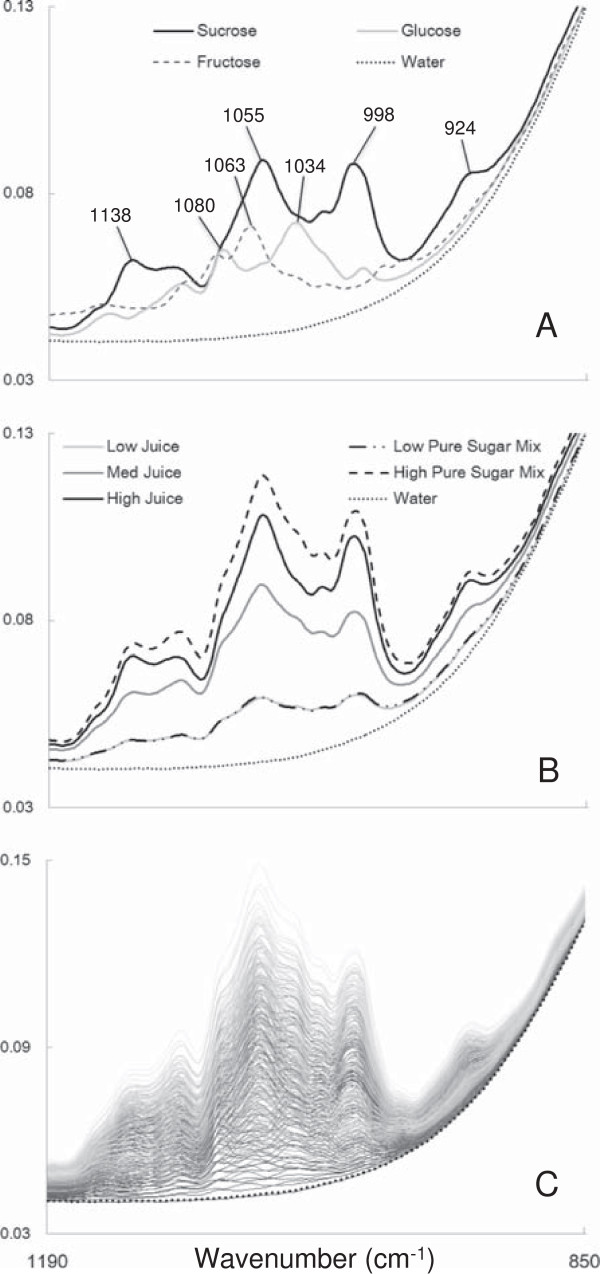
**FTIR spectra of pure sugars, *S. bicolor *juice, and the PLS model calibration set.** FTIR spectral fingerprint region collected from: **(A)** pure sucrose, glucose, fructose, and H_2_O; and **(B)***S. bicolor* juice containing low, medium, and high concentrations of sugars and a low and high mixture of pure sucrose, glucose, and fructose against a H_2_O background. **(C)** Set of 216 samples consisting of varying mixtures of sucrose, glucose, and fructose making up the calibration set. Wavenumbers between 1,190 to 850 cm^-1^ were plotted. X-axis represents spectral wavenumber (cm^-1^) and y-axis units are arbitrary. FTIR, Fourier transform infrared; PLS, partial least squares.

FTIR spectra were collected from pure sucrose, glucose, fructose, and H_2_O samples (Figure 3A) for comparison with *S. bicolor* juice samples that were known to contain low, medium, and high mixtures of sucrose, glucose, and fructose (as determined initially by brix and then gas chromatography–mass spectrometry, GC-MS, data not shown) and low/high concentration mixtures of pure sugars (Figure 3B). Pure sucrose, glucose, and fructose each exhibit unique spectra that were easily identifiable above the consistent H_2_O background (Figure 3A); however, each had overlapping IR signatures making quantification by Beer’s law inappropriate, since the variations of one sugar would affect the IR signature of the others. Multivariate PLS regression was therefore required to generate predictive models for each sugar that would model this accurately.

It was shown that pure sucrose, glucose, and fructose mixtures have almost identical IR signatures in the fingerprint region (specifically from 1,190 to 850 cm^-1^) to that of juice pressed from *S. bicolor* (Figure 3B), not surprisingly, as sucrose, glucose, fructose, and H_2_O are the major constituents of *S. bicolor* juice [26]. Hence, a PLS model for sucrose, glucose, and fructose could justifiably be calibrated using 216 mixtures of pure sucrose, glucose, and fructose as the calibration set (Figure 3C) (see Additional file 4 for concentrations).

Using these 216 calibration mixtures, PLS regression models were generated using the non-linear iterative partial least squares (NIPALS) algorithm (Unscrambler X, Camo, Woodbridge, NJ, USA) with mean centred data and full cross-validation. The FTIR spectral fingerprint region (1,800 to 800 cm^-1^) was used as the x-predictor variables and sucrose, glucose, and fructose concentrations as the y-response variables. It was found that variable reduction (that is, reducing the number of spectral prediction variables) resulted in more robust models. Raw spectra truncated to 1,180 to 900 cm^-1^ wavenumbers were found to give the most accurate models and were used to generate the final PLS models for sucrose, glucose, and fructose. A model with six latent variables (LVs) minimised the root mean squared error of calibration (RMSEC) and maximised *R*^*2*^ for sucrose and fructose giving a RMSEC of 5.62 and 2.46 mM, respectively, and both with *R*^*2*^ of 0.99. A model with nine LVs was selected for glucose, which gave a RMSEC of 2.69 mM and *R*^*2*^ of 0.99. It is not determined why glucose required three additional LVs, however, predictions with six LVs were also accurate. This was not thought to be over-fitting since independent validation samples performed slightly better with nine LVs in comparison to six LVs.

**Figure 4:**
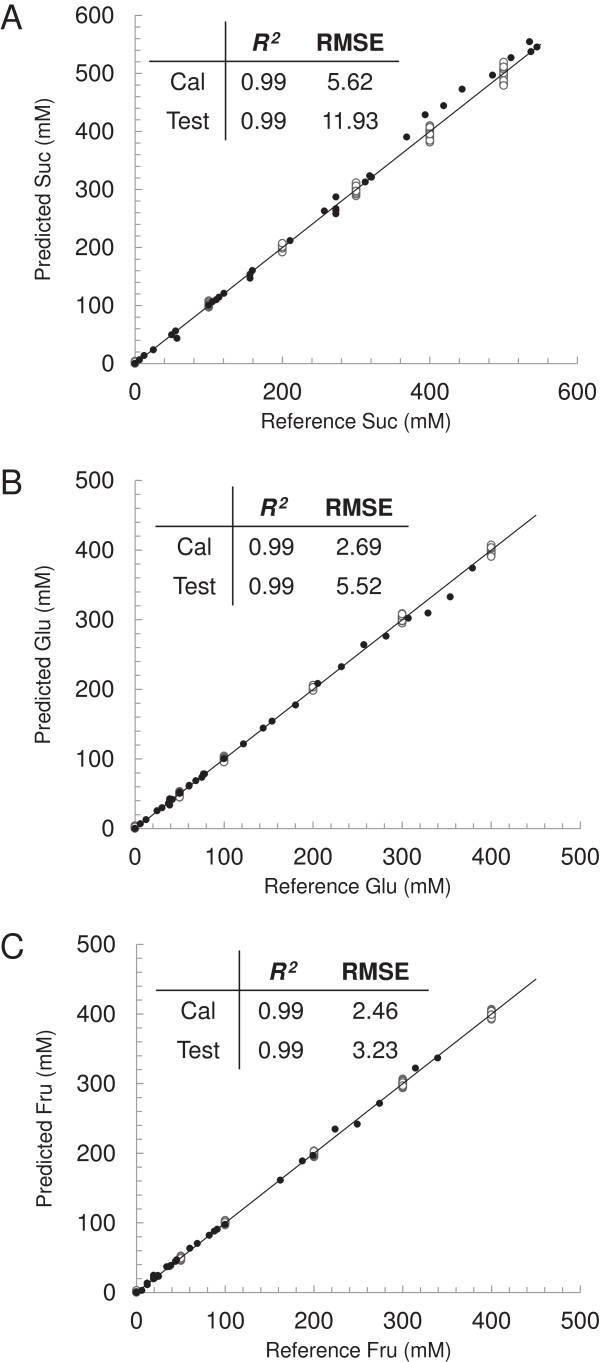
**Soluble sugar FTIR PLS prediction models.** PLS prediction of **(A)** sucrose (mM), **(B)** glucose (mM), and **(C)** fructose (mM) in an independent test set of *S. bicolor* juice mixtures. Predictions are plotted against the known reference values for each sample. Calibration samples (○) and independent validation samples (•) were overlaid on a target line (*y = x*). The coefficient of determination (*R*^*2*^) and root mean square error (RMSE) for each sample set are displayed for each sugar. FTIR, Fourier transform infrared; PLS, partial least squares; RMSE, root mean squared error.

Each PLS model was offset by subtracting the y-intercept of the cross-validation predicted versus reference plot (effectively subtracting the water background) and a small slope adjustment factor was introduced for sucrose to minimise the slope deviation from one of the cross-validation samples. The PLS models, including adjustment factors, were then assessed with an independent test set consisting of three variable *S. bicolor* juice samples of known sugar concentrations, which were either spiked with pure sucrose, glucose, and fructose, separately and mixtures of the three, or diluted to achieve a large variable set of juice samples with known amounts of sugars. This was thought to be a more accurate method for generating a variable test set of juice samples than collecting a large variable set of *S. bicolor* juice samples and determining their sugar concentrations by GC-MS or HPLC. When the predicted values were plotted against the reference values for sucrose (Figure 4A), glucose (Figure 4B), and fructose (Figure 4C), the independent test set achieved a root mean squared error of validation (RMSEV) of 11.93, 5.52, and 3.23 mM, respectively, and a validation coefficient of determination (*Q*^*2*^) of 0.99 for each (*R*^*2*^ is used to denote the coefficient of determination of calibration samples and *Q*^*2*^ is used for validation samples). According to Tamaki and Mazza (2011) [27] these indicate ‘excellent’ models since *Q*^*2*^ >0.9 and *Q*^*2*^ – *R*^*2*^ <0.2, and are certainly adequate for a high-throughput screen. Furthermore, LV coefficient peaks for each sugar corresponded with the major sucrose, glucose, and fructose peaks (Figure 3A) (Additional file 5) indicating that the PLS regression models are indeed based on the chemistry of the sugars. The lower limit of detection (LoD) was conservatively determined to be approximately 25 mM, and spike/dilution tests were shown to be accurate (Additional file 6).

**Figure 5:**
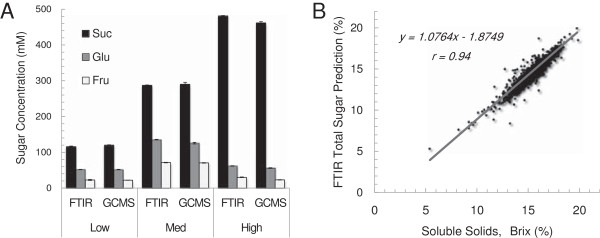
**Comparison of the FTIR PLS model to GC-MS and brix.** PLS prediction of sucrose (mM), glucose (mM), and fructose (mM) compared to values obtained using **(A)** GC-MS and **(B)** total sugars (g/100 mL) calculated using sucrose, glucose, and fructose PLS prediction models compared with total soluble solids (°brix). A linear regression line is fitted to the data and the equation and correlation (*r*) is displayed. FTIR, Fourier transform infrared; GC-MS, gas chromatography–mass spectrometry; PLS, partial least squares.

To further assess the accuracy of the generated PLS models, sucrose, glucose, and fructose were measured in three variable *S. bicolor* juice samples ‘low’ (120 mM sucrose, 52 mM glucose, 22 mM fructose), ‘medium’ (290 mM sucrose, 126 mM glucose, 70 mM fructose), and ‘high’ (461 mM sucrose, 56 mM glucose, 23 mM fructose) with six technical replicates using GC-MS and compared to results obtained with the PLS models (Figure 5A). Results were comparable (Figure 5A). Similarly 1,000 *S. bicolor* juice samples for which brix had been measured were assessed using the high-throughput FTIR-based PLS models. Total sugars (g/100 mL) were obtained by addition of sucrose, glucose, and fructose (g/100 mL) calculated from their respective PLS predictions using molar masses. A correlation of 0.94 between brix and total sugars (g/100 mL) was observed over the range of 5 to 20 g/L with an offset of −1.87 g/100 mL and a slope of 1.08 (Figure 5B). The observed negative offset indicated that brix readings were generally higher than those obtained from the FTIR PLS models. This was to be expected since brix measures not only sugars but all soluble solids in the sample. A slope greater than 1 suggests that this affect is less pronounced at higher sugar levels. This relationship to brix therefore supports the robustness of the PLS prediction models and it is suggested that this could be a more accurate method for widespread adoption by the sugar industry, specifically since it facilitates high-throughput quantification of sucrose, glucose, and fructose separately. In addition, the FTIR method (30 seconds per sample) is much more rapid than GC-MS (1 hour per sample) or HPLC (20 minutes per sample) and requires no sample preparation. *S. bicolor* juice samples have been shown to degrade rapidly at room temperature [26] which means that samples must be treated to remove bacterial contamination and enzyme activity or HPLC devices must have a refrigerated autosampler if long sample runs are to be performed. Since the FTIR PLS prediction method is rapid there are no such issues with degradation of sugars. Whilst FTIR and NIR prediction of juice sugars have been reported previously [17,19,21], to our knowledge, this is the first report of accurate predictions of sorghum juice sugars. Although, based on previous reports, it was expected that models would be accurate, developing them was integral to the implementation of this high-throughput methodology.

To convert the measured sugar concentrations into sugar yield per stalk, *V*_*ratio*_ and the calculated whole stalk FW and DW (thus relative water content) from Section 1 could be used.

### FTIR prediction of cell wall digestibility

Biomass, or more specifically, the plant cell wall, is a complex matrix of polymers; therefore, it was not possible to create a variable calibration set of cell walls in the laboratory, as was done with the soluble sugar PLS models. A selection of 90 highly variable *S. bicolor* ecotypes at varying developmental stages, grown under a range of different conditions (see Additional file 7 for sample descriptions) was, therefore, used to calibrate and validate an FTIR-based cell wall digestibility model.

**Figure 6:**
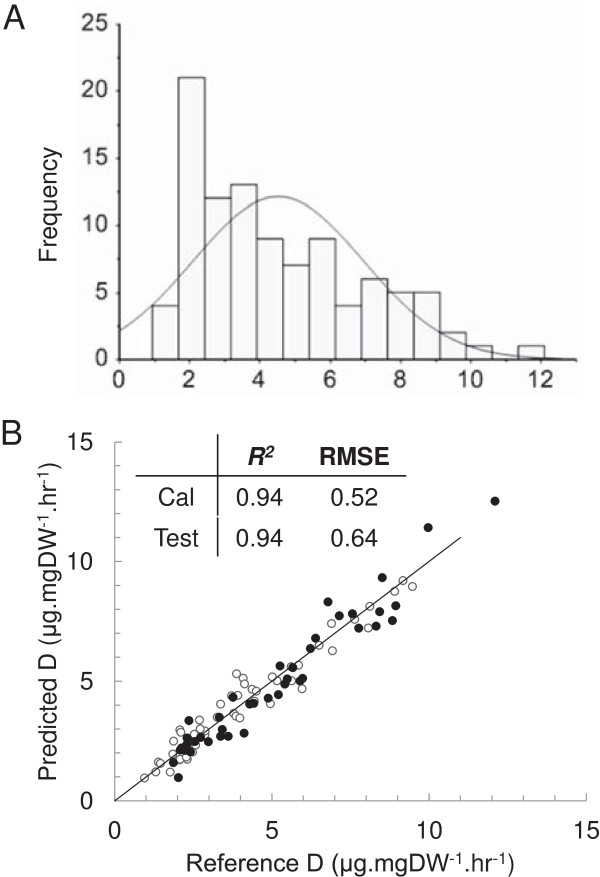
**FTIR prediction of cell wall digestibility. (A)** Distribution of cell wall digestibility (μg.mgDW^-1^.h^-1^) from a diverse collection of *S. bicolor* ecotypes grown under a range of different conditions and harvested at varying developmental stages. A fitted normal distribution has been overlaid. **(B)** PLS prediction of cell wall digestibility (*D*; μg.mgDW^-1^.h^-1^) in an independent test set. Predictions are plotted against the known reference values for each sample. Calibration samples (○) and independent validation samples (•) were overlaid on a target line (*y = x*). The coefficient of determination (*R*^*2*^) and root mean square error (RMSE) for each sample set are inset. DW, dry weight; FTIR, Fourier transform infrared; PLS, partial least squares; RMSE, root mean squared error.

Digestibility (*D*) was quantified by enzymatic hydrolysis of ball-milled cell walls using a *Trichoderma reesei* cellulase mixture (Sigma-Aldrich, Castle Hill, Australia) and the released reducing sugars were measured by the 3,5-dinitrosalicylic acid (DNS) method [2]. A large range of digestibility was observed from 0.95 to 12.1 μg.mgDW^-1^.h^-1^ with the majority of samples lying between 1 and 10 μg.mgDW^-1^.h^-1^ (Figure 6A). From this variable set of samples, 52 were selected as the calibration set and 38 as the independent validation set so as to approach a uniform distribution between 1 and 10 μg.mgDW^-1^.h^-1^ as closely as possible for each set.

FTIR spectra were collected from the selected calibration set and a PLS regression model for cell wall digestibility was generated using second derivative spectra with an extended multiplicative scatter correction (EMSC) applied (spectra available in Additional file 8). A PLS model with spectra truncated to 1,800 to 850 cm^-1^ was calibrated and regression coefficients with 90% confidence intervals that spanned 0 were removed (that is, insignificant variables). The final PLS prediction model was calibrated from 147 wavenumbers with four LVs minimising the RMSEC (0.52 μg.mgDW^-1^.h^-1^) and maximising the *R*^*2*^ (0.94) (Figure 6B). The model was then used to predict the digestibility of an independent test set.

When the predicted values of the independent test set were plotted against the reference (as determined by DNS assay) a RMSEV of 0.64 μg.mgDW^-1^.h^-1^ and *Q*^*2*^ of 0.94 were observed (Figure 6B). This indicated an ‘excellent’ model since *Q*^*2*^ >0.9 and *Q*^*2*^ – *R*^*2*^ <0.2 [27], and is adequate for a high-throughput screen. In addition, the PLS model regression coefficients resembled cell wall spectral peaks (Additional file 9) indicating that the model was based on cell wall chemistry. Analysis of the FTIR spectral peaks contributing to the prediction of cell wall digestibility in the PLS model could elucidate novel cell wall chemistry that is related to digestibility, however, this was outside the scope of this methods paper.

**Figure 7:**
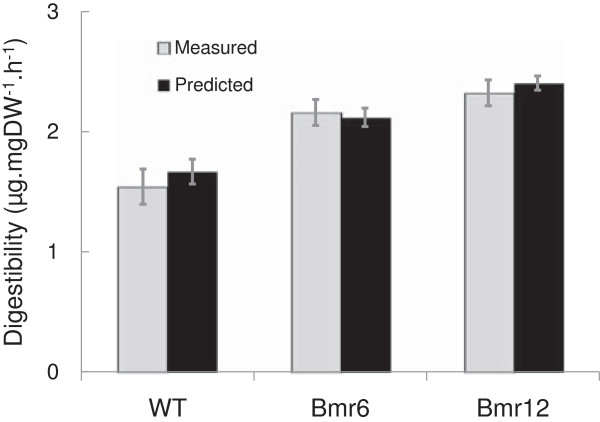
**FTIR PLS prediction of cell wall digestibility in *S. bicolor *mutants.** PLS prediction of cell wall digestibility (μg.mgDW^-1^.h^-1^) in *S. bicolor* mutants *bmr6*, *bmr12*, and their parent line (WT) harvested at 10 days post-anthesis, compared with DNS determination of digestibility. Data are represented as means of six biological replicates ± SE. DNS, 3,5-dinitrosalicylic acid; DW, dry weight; FTIR, Fourier transform infrared; PLS, partial least squares; SE, standard error.

To further assess the PLS digestibility model, the digestibility of *S. bicolor* mutants, *bmr6*, *bmr12*, and their parent line was measured (six biological replicates) using both DNS assay and the FTIR-based PLS model (Figure 7). An increase in digestibility in both *bmr6* and *bmr12* was observed, as previously reported in the literature [28-30], and both methods produced comparable values (Figure 7). The observed biological variation (standard error), was also comparable between the two methods (Figure 7) providing further validation of the PLS prediction of digestibility.

To convert the measured digestibility into cell wall hydrolysis yield per stalk, the calculated stalk DW (using *V*_*ratio*_ from Section 1) could be used, assuming a 24-hour cellulase digestion.

FTIR prediction of digestibility by this method is much more rapid (approximately 45 seconds per sample) than cellulase digestion, which requires a 24-hour incubation and an average total processing time (excluding incubations) of approximately 15 minutes per sample. FTIR prediction of enzymatic cell wall hydrolysis has previously been demonstrated [16], as has biomass analysis by NIR spectroscopy in *Miscanthus giganteus*[22], switchgrass [31], and corn stover [32]; however, this is the first example in *S. bicolor* and the first time these predictive models have been incorporated into a holistic high-throughput screen in a biofuels context. FT-MIR was advantageous in our high-throughput screen since MIR spectra were used in the prediction of soluble sugars, thus only one Portable IR machine was required. MIR cell wall spectral peaks are also more readily interpreted than NIR spectral peaks [10], which allowed downstream interpretation of cell wall chemistry in variants identified from the high-throughput screen, but is beyond the scope of this publication.

It should also be noted that each PLS predictive model is specific for the wet chemistry method employed. In our case, a *T. reesei* cellulase mixture was used on biomass subjected to milling followed by a weak acid pre-treatment under 100 KPa pressure, a 24-hour incubation at pH 4.8/55°C, and the resulting reducing sugars measured by the DNS assay. Currently, biomass composition data as determined by the National Renewable Energy Laboratory (NREL) method [33] is being used to calibrate PLS models from the exact same spectral and biomass sample set. This highlights the fact that numerous wet chemistry and hydrolysis methodologies can be applied to the same variable sample set to calibrate numerous PLS predictive models. Prediction of biomass composition and enzymatic hydrolysis under varying conditions could then be made simultaneously from a single spectra. Once plants are being grown specifically for lignocellulosic ethanol production, this technique could be applied in an agricultural context to dictate price per ton based on a predicted hydrolysis yield that is unique to the methods employed by each processing plant.

### Calculation of total fermentable sugars

**Figure 8:**
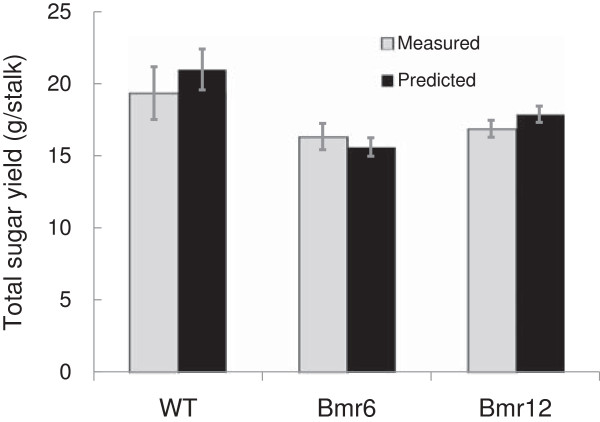
**Calculated total fermentable sugar yield in *S. bicolor *mutants.** Combined total sugar yield from saccharification of the cell wall fraction and soluble sugar fraction in *S. bicolor bmr6* and *bmr12* mutants harvested at 10 days post-anthesis as determined by wet chemistry of whole stalks (measured) and FTIR predictive models extrapolated from the fourth internode (predicted). Data are represented as means of six biological replicates ± SE. FTIR, Fourier transform infrared; SE, standard error.

Using the equation:

$$V.\left[S\right]+B.D=\mathrm{total}\;\mathrm{fermentable}\;\mathrm{sugar}\;\mathrm{yield}$$

where whole stalk juice volume (*V*) and dry weight of biomass (*B*) were calculated in Section 1, total sugar concentration ([*S*]) was determined in Section 2, and digestibility (*D*) was determined in Section 3, the total fermentable sugar content of *bmr6*, *bmr12*, and their parent line (WT) were calculated (Figure 8). The high-throughput methodology was compared to values obtained using traditional methods (that is, brix and DNS cellulase assay) on whole stalks (see Additional file 10 for whole stalk yield equations). Both methods produced comparable results with comparable standard errors (Figure 8) showing the accuracy and robustness of the holistic high-throughput screening methodology. In addition, *bmr6* and *bmr12* mutations have been reported numerous times to result in a yield reduction of 15% to 30% [30,34], which is consistent with the observed total fermentable yield reduction of 19% and 16% in *bmr6* and *bmr12*, respectively (Figure 8).

Using this holistic high-throughput methodology for screening large variable biofuel feedstock populations, once samples were oven dried, three workers with very minimal training were able to process approximately 200 samples per day which encompassed harvesting through to a total fermentable sugar yield calculation. Data on cell wall composition, stalk sucrose, glucose, and fructose concentrations, relative water content, and stalk geometry including plant height was collected in the process. In addition, the majority of the processing time was consumed in the pressing and grinding of internodes, thus automation of these processes has the potential to dramatically increase throughput. The methodology was, however, designed to be transportable to any field site or laboratory, thus the use of non-portable, specialised, or expensive equipment was avoided. In comparison, using a traditional wet chemistry approach, and once samples were accumulated through multiple processes including oven drying and numerous incubation steps, three workers with moderate laboratory skills could process only approximately ten samples per day without collecting any information on cell wall composition or plant geometry. The traditional method required a laboratory set-up and was not easily portable to field sites.

## Conclusion

FTIR spectral prediction models have been used in the food industry to predict sucrose, glucose, and fructose in fruit and sugarcane juice [17-19] and, more recently, to predict cellulase digestibility of lignocellulose [16]. Here we present a high-throughput screening methodology that unifies and simplifies previous methods to produce a holistic assessment of biofuel feedstock potential via a total fermentable sugar yield calculation. In the process, a wealth of information on cell wall composition, the soluble sugar fraction, and plant geometry was generated rapidly using equipment that is relatively cheap, accessible, easily operated, and transportable to any field site.

## Materials and methods

### Plant growth conditions

One hundred sweet *S. bicolor* ecotypes were field grown at the Pacific Seeds Gatton Research Centre (Queensland, Australia) in September 2009 and were harvested in February 2010 approximately 10 days post-anthesis. Fifteen genotypes were selected as possible cell wall digestibility variants based on physical characteristics such as stalk strength, stiffness, and leaf midrib colour which were determined subjectively. Subsequently, seed from these 15 selected genotypes, along with the sweet sorghum variety ‘Rio’, were sown in a glasshouse in Newcastle, Australia, in May 2010 in 25 cm diameter pots. Plants in Newcastle were supplemented with 300 μmol.m^-2^.s^-1^ photosynthetically active radiation (PAR; 400 to 700 nm) from 4:00 pm to 6:30 pm each day and natural sunlight throughout the winter growth period ranged from 200 to 1,100 μmol.m^-2^.s^-1^ PAR with 28°C/15°C day/night temperatures. Plants were harvested in August 2010. This provided a set of phenotypically variable plants from which predictive models were built. The correlation studies were performed on 15 proprietary sweet sorghum cultivars that were field grown during the 2009 planting at the Pacific Seeds Gatton Research Centre. Descriptive data for these 15 lines is available in Additional file 3. Cell wall digestibility correlations were supplemented with four replicate glasshouse grown ‘Rio’ plants to replace lost samples.

### Biomass assessment

A new high-throughput methodology was developed for screening large variable populations of *S. bicolor* for biomass, soluble sugar concentrations, and cell wall composition using a volumetric correlation between the fourth internode from the base of the stalk that had expanded more than 2 cm, to that of the whole stalk (Figure 1). During harvest of the fourth internode four field measurements were taken: stalk height (*H*), stalk base diameter (*R*), stalk top diameter (*r*), and length from the base of stalk to the base of the fourth internode (*H*_*B*_). Each internode was cooled then transported to the field station where internode length (*L*_*SI*_) and internode FW were recorded. Each internode was pressed using a Sukra Sugarcane Crusher (S.A. Ivy Multi Pumps Limited, Tamil Nadu, India) and FTIR spectra acquired. Two mL of juice was frozen and stored at −20°C. Pressed *S. bicolor* bagasse was bagged, dried, weighed, and ground. For correlations analysis, whole stalks were stripped of leaves, weighed, pressed, dried, and ground. See Additional file 1 for detailed protocol.

### Fourier transform infrared (FTIR) spectroscopy of soluble sugars

Juice samples were centrifuged at 10,000 *g* for 30 seconds before 50 μL was placed onto a Spectrum II (PerkinElmer, Waltham, MA, USA) with a universal diamond ATR attachment operated by Spectrum One software package (PerkinElmer). Absorbance spectra were collected at a resolution of 4 cm^-1^ over two scans from 4,000 to 400 cm^-1^ wavenumbers. Spectra were imported into Unscrambler X 10.0.1 (Camo) and truncated to the fingerprint region. Predictive models were optimised when variable reduction was employed to further truncate spectra to 1,180 to 900 cm^-1^ wavenumbers. A 9-point Savitzky–Golay smoothing algorithm was applied to juice spectra from both the calibration and validation sets (see Additional file 4 for sample catalogues) before model calibration and prediction.

### Gas chromatography–mass spectrometry (GC-MS) of soluble sugars

Eleven μL of sorghum juice, plus 5 μL of a 2 mg mL^-1^ ribitol internal standard was lyophilised. Derivatisation/trimethylsilylation and quantification of sugars by GC-MS were performed following the published procedure [35] with the following modifications. The GC-MS system used was a 5973A MSD (Agilent Technologies, Santa Clara, CA, USA), GC was performed on a 30 m BPX5 column (SGE, Victoria, Australia), and both chromatograms and mass spectra were evaluated using the Enhanced MSD ChemStation D.01.02 software (Agilent Technologies). Mass spectra of eluting sugars were identified using the Wiley Registry of Mass Spectral Data (Wiley-Blackwell, Hoboken, NJ, USA), and all compounds were verified by subsequent analysis of pure standards. Absolute quantification of sugars was achieved by equating normalised areas to a set of standard curves for sucrose, glucose, and fructose.

### Cell wall preparation

Dried *S. bicolor* bagasse (pressed internode) was ground to a fine powder (<100 μm) with a TissueLyser II (Qiagen, Venlo, Netherlands) at a frequency of 1/30 for 2 minutes using 1.5 cm diameter stainless steel ball bearings. A total of 150 mg of powder was then washed with 1.7 mL of water and dried in a vacuum concentrator centrifugal evaporator (Jouan RC 10.10, St. Herblain, France) before acquiring FTIR spectra. For model calibration and validation, a series of extraction steps following the published procedure [36] was performed on 120 mg of washed powder before determination of cell wall saccharification efficiency by enzymatic hydrolysis.

### FTIR spectroscopy of cell walls

Approximately 50 mg of washed and dried cell wall material was mounted onto a Spectrum II (PerkinElmer) with a universal diamond ATR attachment operated by Spectrum One software package. The cell wall powder was forced onto the ATR crystal with a press at a constant force. Spectra were collected at a resolution of 4 cm^-1^ and co-added over three scans. Once all spectra were collected, data was imported into Unscrambler X 10.0.1 (Camo) for pre-treatment. Spectra were truncated to the cell wall fingerprint region (1,800 to 850 cm^-1^ wavenumbers), second derivative spectra were calculated by the Savitzky–Golay method with nine smoothing points and an EMSC was applied to correct for light scattering by cell wall particles.

### 3,5-Dinitrosalicylic acid (DNS) determination of cell wall saccharification

Fifteen mg of isolated cell wall was vacuum infiltrated under 100 KPa pressure for 5 minutes in 1.25 mL of 50 mM sodium citrate buffer (pH 4.8) followed by addition of 50 μL of 1 mg/mL *T. reesei* cellulase mixture (Sigma-Aldrich) and a control was included for each sample where cellulase was replaced with buffer. Samples were vortexed briefly and incubated at 55°C with gyrorotation at 250 rpm for 24 hours. Cellulase digested material was centrifuged at 3,500 *g* for 3 minutes. One mL of supernatant was added to 3 mL of DNS reagent and incubated for 15 minutes in a 100°C water bath. One mL of 40% (w/v) potassium tartrate and 20 mL of dH_2_O was immediately added to dilute and stabilise the colour. Absorbance was measured at 540 nm and compared to a D-glucose standard curve to determine the total amount of reducing sugars.

### Partial least squares (PLS) model calibration and validation

Pre-treated spectra were imported into the Unscrambler X 10.0.1 (Camo) for data analysis. The soluble sugar predictive model used 216 sugar standards to calibrate a PLS regression using the NIPALS algorithm, and the cell wall digestibility predictive model used 58 hand-picked calibration samples (that were representative of the range in digestibility) with the ‘wide-kernel PLS’ algorithm [37]. All models used mean centred data, computed a default of 15 factors, and used full cross-validation. Outliers were identified using residual and leverage plots and were manually examined to ensure that they were outliers based on technical errors in spectral collection or wet chemistry before removal, and then PLS algorithms were re-calculated. The optimal number of factors was chosen as that which minimised the RMSEV and maximised the amount of sample variation that was described by the model. The performance of models must be continually checked against wet chemistry and continually updated with biological extremes to increase the range of samples accurately predicted by the model, therefore samples that lie outside of the range of the model should be analysed and incorporated into the calibration set. The models presented here are, therefore constantly being updated and improved as more samples are screened and this is an integral part of the methodology of chemometric model development.

PLS models were validated by prediction on an independent test set of 33 soluble sugar samples and 25 cell wall samples (see Additional files 4 and 7 for calibration and validation sample catalogues). Predicted values were plotted against reference values and the RMSEP and validation coefficient of determination (*Q*^*2*^) were used to assess the accuracy of the models. The analysis of *bmr6*, *bmr12*, and their parent line (WT) was performed independently using the methods outlined above.

## Abbreviations

ATR: Attenuated total reflectance; Bmr: Brown midrib; DNS: 3,5-Dinitrosalicylic acid; DW: Dry weight; EMSC: Extended multiplicative scatter correction; FIR: Far-infrared; FTIR: Fourier transform infrared; FT-MIR: Fourier transform mid-infrared; FW: Fresh weight; GC-MS: Gas chromatography–mass spectrometry; HPLC: High-performance liquid chromatography; IR: Infrared; LoD: Limit of detection; LV: Latent variable; MIR: Mid-infrared; NIPALS: Non-linear iterative partial least squares; NIR: Near-infrared; NREL: National renewable energy laboratory; PAR: Photosynthetically active radiation; PDA: Personal digital assistant; PLS: Partial least squares; RMSEC: Root mean squared error of calibration; RMSEP: Root mean squared error of prediction; RMSEV: Root mean squared error of validation; WT: Wild type.

## Competing interests

The authors declare that they have no competing interests.

## Authors’ contributions

APM theorised the sugar and cell wall FTIR models, and collaboratively theorised the internode correlation study with WP. APM designed all experiments, collected all data, analysed all data, produced Figures 1, 2, 3, 4, 5, 6, 7, and 8, and drafted the manuscript. WP designed and collaborated on the sugar model data collection and analysis, including volumetric internode correlation experiments, and produced Figures 1, 2, 3, 4, and 5. CB reviewed experimental design and provided feedback on the digestibility model. CPLG conceived and established the research program and contributed to overall experimental design. RTF contributed to experimental design and reviewed the manuscript. All authors read and approved the final manuscript.

## Supplementary Material

Additional file 1 **High-throughput screening protocol.** A detailed step-by-step protocol for performing the described high-throughput screening platform. Click here for file

Additional file 2 **Volumetric ratio derivation.** A mathematical derivation of the volumetric ratio used to relate measurements on the fourth internode to whole stalks. Click here for file

Additional file 3 **Sweet sorghum lines used for correlation analysis.** Fresh weight (FW), dry weight (DW), and height data for the 15 sweet sorghum lines used in the correlations analysis. DW, dry weight; FW, fresh weight. Click here for file

Additional file 4 **Calibration and validation samples used for the sugar PLS models.** Sucrose, glucose, and fructose content of the calibration and validation sample sets used for calibrating and validating the sugar FTIR PLS models. FTIR, Fourier transform infrared; PLS, partial least squares. Click here for file

Additional file 5 **Sugar model regression coefficients.** Weighted regression coefficients from the sucrose, glucose, and fructose PLS models showing that regression coefficients resemble pure standard spectra of each sugar. PLS, partial least squares. Click here for file

Additional file 6 **Spike/dilution recovery and limit of detection of the FTIR PLS sugar models.** Limit of detection and spike/dilution recovery of sucrose, glucose, and fructose using the FTIR PLS models for each sugar. FTIR, Fourier transform infrared; PLS, partial least squares. Click here for file

Additional file 7 **Calibration and validation samples used for the digestibility PLS model.** Sorghum lines and sampled tissue used for calibrating and validating the digestibility PLS model. PLS, partial least squares. Click here for file

Additional file 8 **Processed spectra used to calibrate the digestibility PLS model.** Second derivative spectra with an EMSC applied, which were used to calibrate the digestibility PLS model. EMSC, extended multiplicative scatter correction; PLS, partial least squares. Click here for file

Additional file 9 **PLS digestibility model diagnostics and band assignment chart.** Model diagnostics for the PLS digestibility model showing clear separation of digestibility in the scores plot and a representation of cell wall peaks in the regression coefficients. A band assignment chart is displayed for reference. PLS, partial least squares. Click here for file

Additional file 10 **Whole stalk fermentable sugar calculations.** Whole stalk calculations for fermentable sugars in the soluble sugar fraction and cell wall fraction resulting in a total fermentable sugar yield calculation. Click here for file
