# Supplementary material for: A holistic high-throughput screening framework for biofuel feedstock assessment that characterises variations in soluble sugars and cell wall composition in Sorghum bicolor
Source: Biotechnol Biofuels. 2013 Dec 23;6:186. doi: 10.1186/1754-6834-6-186 (PMC3892131; doi:10.1186/1754-6834-6-186)
Supplement: Additional file 1 — High-throughput screening protocol. A detailed step-by-step protocol for performing the described high-throughput screening platform. [file 1754-6834-6-186-S1.pdf]

Sweet Sorghum Field Harvesting Protocol: Biofuel feedstock screening

1. Field harvest

Materials and equipment:

Secateurs
Marker pen
Personal digital assistant (PDA) #smart phone is sufficient
4m ruler
Electronic callipers
Insulated cooler
5 mL sealable tubes
Waxed paper bags (optional)
Stapler (optional)

Protocol:

This protocol can be completed individually however, two people working together is optimal.

1) Cut main stalk at ground level.
2) Lay stalk down on ground and measure total plant height to the base of the seed head (**Figure 1A** and **B**) and length from the base of the stalk to the base of the 4th internode (**Figure 1C**).
3) Cut out 4th internode, remove leaf sheath then record sample number on the internode with permanent marker before placing immediately into a cold insulated cooler (**Figure 1D**).

   ## Longer than 2hrs of storage at room temperature can result in significant enzymatic hydrolysis of sucrose.

4) Measure top and bottom diameters with electronic callipers.

Record all data directly into a PDA which can be immediately downloaded upon completion and once insulated cooler is full, proceed rapidly to laboratory processing to minimise enzymatic hydrolysis of sucrose.

Optional: Count the number of tillers 50cm either side of the harvested tiller as a measure of tiller density. Numerous additional measures are suggested including developmental stage, the amount of lodging and other phenotypic anomalies.

Notes:

Insulated cooler set-up: A 2-wheel insulated cooler was used so that excised internodes could be immediately kept at ~0°C. A rubber slot was cut into the insulated cooler through which internodes were inserted to reduce heat exchange as it was no longer necessary to open the insulated cooler lid for each sample, and

was a more time efficient method of transferring internodes. Small tubes containing 4 ml of water (frozen) were used instead of ice to rapidly cool internodes since melted ice water can affect relative water contents of internodes.

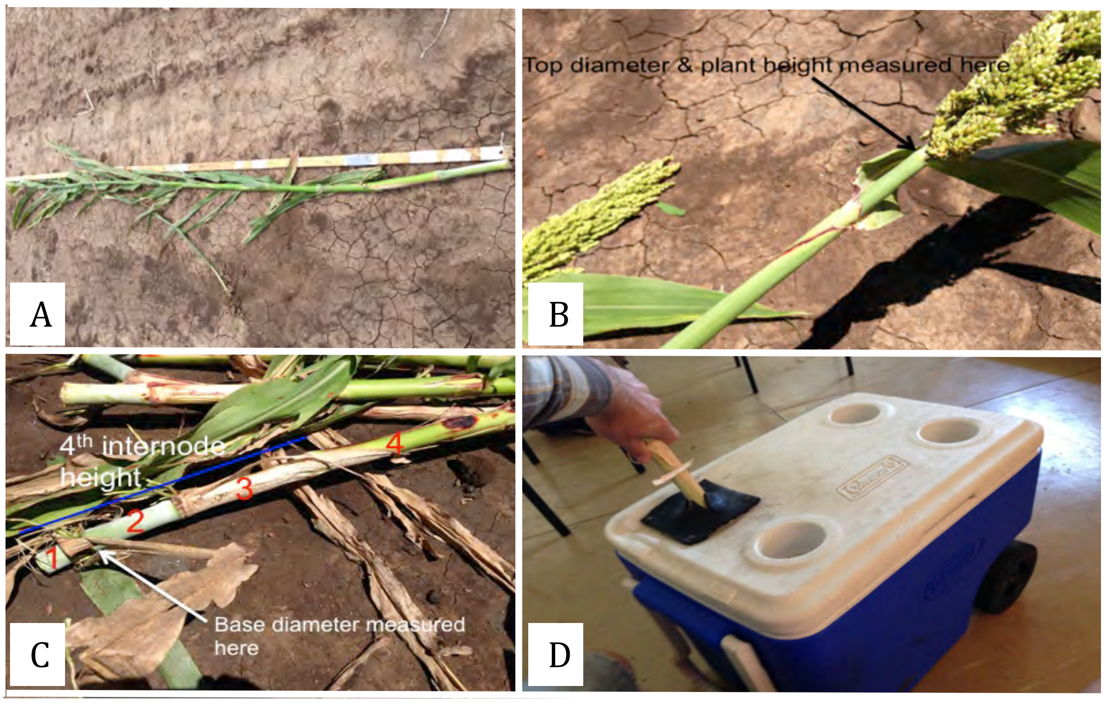

Figure 1. Field harvesting protocol

2. Laboratory processing

Materials and equipment:

Secateurs
Ice packs
-20°C freezer
2 mL and 5 mL tubes
30 cm ruler
Electronic scales
Sukra sugarcane juicer (or equivalent)
Paper sterilisation bags
Drying oven
Spice grinder

Protocol:

1) Place harvested internodes in order between two frozen ice packs (**Figure 2-1**).
2) Measure length of the internode and fresh weight (**Figure 2-2** and **3**).
3) Process internode through juicer (**Figure 2-4**).
4) Collect **ALL** juice (wait for the drips to stop and wipe juicer between each internode) (**Figure 2-5**).

5) Mix juice well, sample 2 mL of juice and collect FTIR spectra immediately see **section 3**). #Alternatively juice can be frozen immediately and stored at -80°C.
6) Place sorghum bagasse in a paper sterilisation bag (**Figure 2-6**).
7) Dry bagasse in drying oven at 70°C until it is completely dry (**Figure 2-7**).
8) Weigh dried bagasse.
9) Grind whole, dried internode to a homogenous, fine powder using a commercial spice grinder or equivalent (**Figure 2-8**).
10) Homogenise ground sample and collect in a 5 mL tube (**Figure 2-9**).

Notes:

Customised juicer insert: A customised flat aluminium tray insert was made and retro fitted in order to minimise juice carryover between samples.

Sampling: Juice samples were processed or frozen immediately to prevent enzymatic hydrolysis of sucrose. Bagasse was immediately dried to prevent fungal growth and care was taken to homogenise the whole ground internode before sampling.

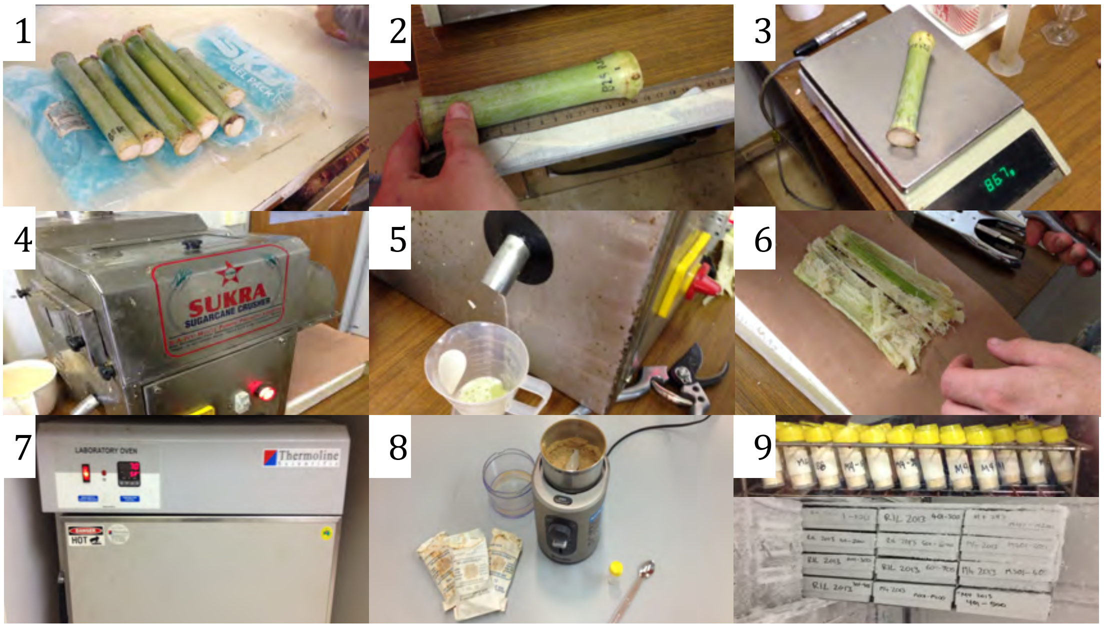

Figure 2. Laboratory processing protocol

3. FTIR spectral acquisition

Materials and equipment:

Transfer pipette
MilliQ H_2O
Heating block
Spatula
Spectrum II with universal diamond ATR accessory (Perkin Elmer) - or equivalent
Spectrum I Software (Perkin Elmer)

Protocol:

Cell Wall -

1) Place ~100 mg of ground sorghum bagasse and collect in a 2 mL eppendorf.
2) Add 1.5 mL of Milli-Q H_2O, vortex thoroughly and heat at 70°C for 10 min.
3) Centrifuge at 10000 g for 1 min to pellet cell wall material.
4) Remove supernatant and dry pellet using a SpeedyVac™ at 40°C or heating block with tubes left open.
5) Place ~50 mg of dried cell wall material onto the FTIR ATR crystal and apply force gauge consistently (to 100% on the Perkin Elmer Spectrum II).
6) Acquire FTIR spectra from 4 scans between 1800-800 cm^{-1}.

Juice -

1) Pipette ~80 µL of juice onto the FTIR ATR crystal.
2) Acquire FTIR spectra from 4 scans between 1800-800 cm^{-1}.

If juice has been frozen for storage: thaw, vortex and centrifuge at 10000 g before acquiring spectra.

4. Prediction models

Materials and equipment:

The Unscrambler X software (Camo Inc.)

Protocol:

1) Import spectra into The Unscrambler X (Camo Inc.) and run prediction models, including spectral pre-processing as described in the main text.
   ## Any other software package capable of generating PLS models can be interchanged here (i.e. R, Matlab etc.).
