# Supplementary material for: A holistic high-throughput screening framework for biofuel feedstock assessment that characterises variations in soluble sugars and cell wall composition in Sorghum bicolor
Source: Biotechnol Biofuels. 2013 Dec 23;6:186. doi: 10.1186/1754-6834-6-186 (PMC3892131; doi:10.1186/1754-6834-6-186)
Supplement: Additional file 2 — Volumetric ratio derivation. A mathematical derivation of the volumetric ratio used to relate measurements on the fourth internode to whole stalks. [file 1754-6834-6-186-S2.docx]

Volumetric Ratio Derivation

The volumetric ratio (V_ratio_) is the ratio of a sampled section of a conical frustum to the whole conical frustum in terms of volume. In our case, we have approximated a *Sorghum bicolor* stem as a conical frustum and from this volumetric ratio we are able to extrapolate data collected from a sampled section of stalk to the whole stem. This relies upon the assumption that the concentration, or density, of the measured value is constant throughout the stalk, or at a fixed ratio from the sampled section to the whole stalk.

**Figure 1.** *Schematic diagram representing a central cross-section of a S. bicolor stalk with the sampled internode/s coloured orange. Large black font represents parameters that were measured, and small blue font represents parameters that were derived. These geometric relationships were used to derive the volumetric ratio between the sample internode/s and the whole stalk (V_ratio_).*

To derive the volumetric ratio between the sampled internode/s and the whole stalk (*V_ratio_*), it is assumed that the whole stalk approximates a conical frustum and the volume of the whole stalk (*V_WS_*) is calculated using the following equation:

$$V_{WS}=\frac{\pi H}{3}\left( r^{2}+rR+R^{2} \right)$$

Where *H* is the height of the stalk, *r* is the radius at the top of the stalk, and *R* is the radius at the bottom of the stalk. These parameters are measured in the field.

Next, the volume of the sampled internode/s (*V_SI_*) must be calculated, however, to do this the top radius (*r_SI_)* and bottom radius (*R_SI_*) of the sampled internode/s must first be derived as follows:

Firstly, an isosceles trapezium from a centred cross section of the conical frustum that represents the whole stalk is taken (see figure 1).

Using simple geometry it can be determined that

$R_{SI}=R-x_{3}$ (see figure 1)……………………………………………………Equation 1.

$\theta=\tan^{-1} \frac{H}{x_{1}}$ …………………………………………………………………………Equation 2.

(since $\theta=\tan^{-1} \frac{opposite}{adjacent}$ from the right angled triangle with sides *H* and $x_{1}$ )

and $\tan\theta=\frac{H_{B}}{x_{3}}$ ………………………………………………………………………Equation 3.

So, substituting equation 2 into equation 3 for $\theta$ gives:

$$x_{3}=\frac{H_{B}}{\tan\tan^{-1} \frac{H}{x_{1}}}$$

$$=\frac{H_{B}x_{1}}{H}$$

$=\frac{H_{B}\left( R-r \right)}{H}$ since $x_{1}=R-r$ (see figure 1)

$\therefore$ substituting into equation 1 gives

$$R_{SI}=R-\frac{H_{B}(R-r)}{H}$$

and by the same logic

$$r_{SI}=R-\frac{(H_{B}+L_{SI})(R-r)}{H}$$

So, since we now know *R_SI_* and *r_SI_*, in terms of parameters that were measured in the field, and since *L_SI_* was also measured, the volume of the sampled internode/s can now be calculated using the equation for a conical frustum as follows:

$$V_{SI}=\frac{\pi L_{SI}}{3}\left( R_{SI}^{2}+r_{SI}R_{SI}+r_{SI}^{2} \right)$$

So, to calculate the volumetric ratio (*V_ratio_*) we simply divide *V_SI_* by *V_WS_*

$$V_{ratio}=\frac{V_{SI}}{V_{WS}}$$
