# Supplementary material for: A holistic high-throughput screening framework for biofuel feedstock assessment that characterises variations in soluble sugars and cell wall composition in Sorghum bicolor
Source: Biotechnol Biofuels. 2013 Dec 23;6:186. doi: 10.1186/1754-6834-6-186 (PMC3892131; doi:10.1186/1754-6834-6-186)
Supplement: Additional file 5 — Sugar model regression coefficients. Weighted regression coefficients from the sucrose, glucose, and fructose PLS models showing that regression coefficients resemble pure standard spectra of each sugar. PLS, partial least squares. [file 1754-6834-6-186-S5.docx]

Sugar model regression coefficients


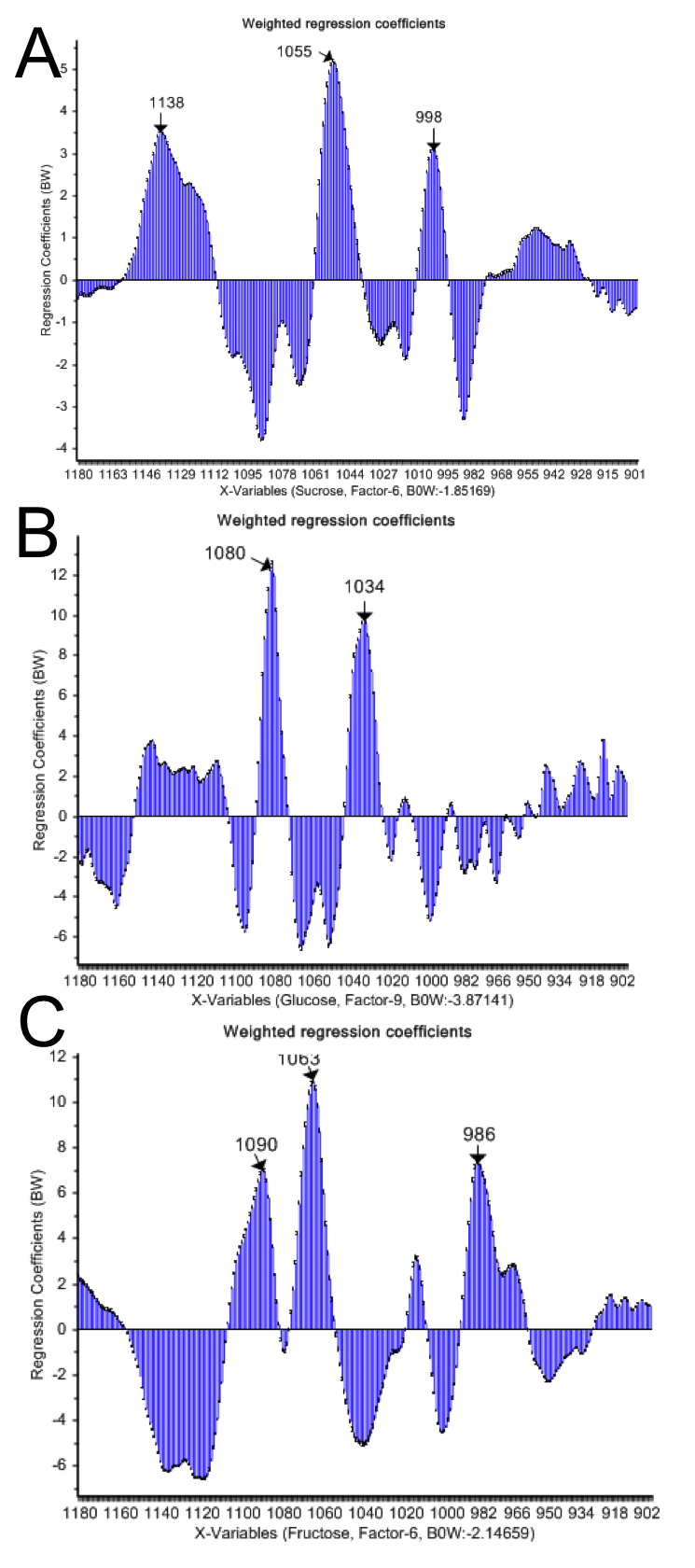


**Figure 1.** Weighted regression coefficients of PLS prediction models developed for sucrose (**A**), glucose (**B**), and fructose (**C**). Peaks corresponding to pure spectra for each sugar are indicated with arrows.
