# Supplementary material for: A holistic high-throughput screening framework for biofuel feedstock assessment that characterises variations in soluble sugars and cell wall composition in Sorghum bicolor
Source: Biotechnol Biofuels. 2013 Dec 23;6:186. doi: 10.1186/1754-6834-6-186 (PMC3892131; doi:10.1186/1754-6834-6-186)
Supplement: Additional file 6 — Spike/dilution recovery and limit of detection of the FTIR PLS sugar models. Limit of detection and spike/dilution recovery of sucrose, glucose, and fructose using the FTIR PLS models for each sugar. FTIR, Fourier transform infrared; PLS, partial least squares. [file 1754-6834-6-186-S6.docx]

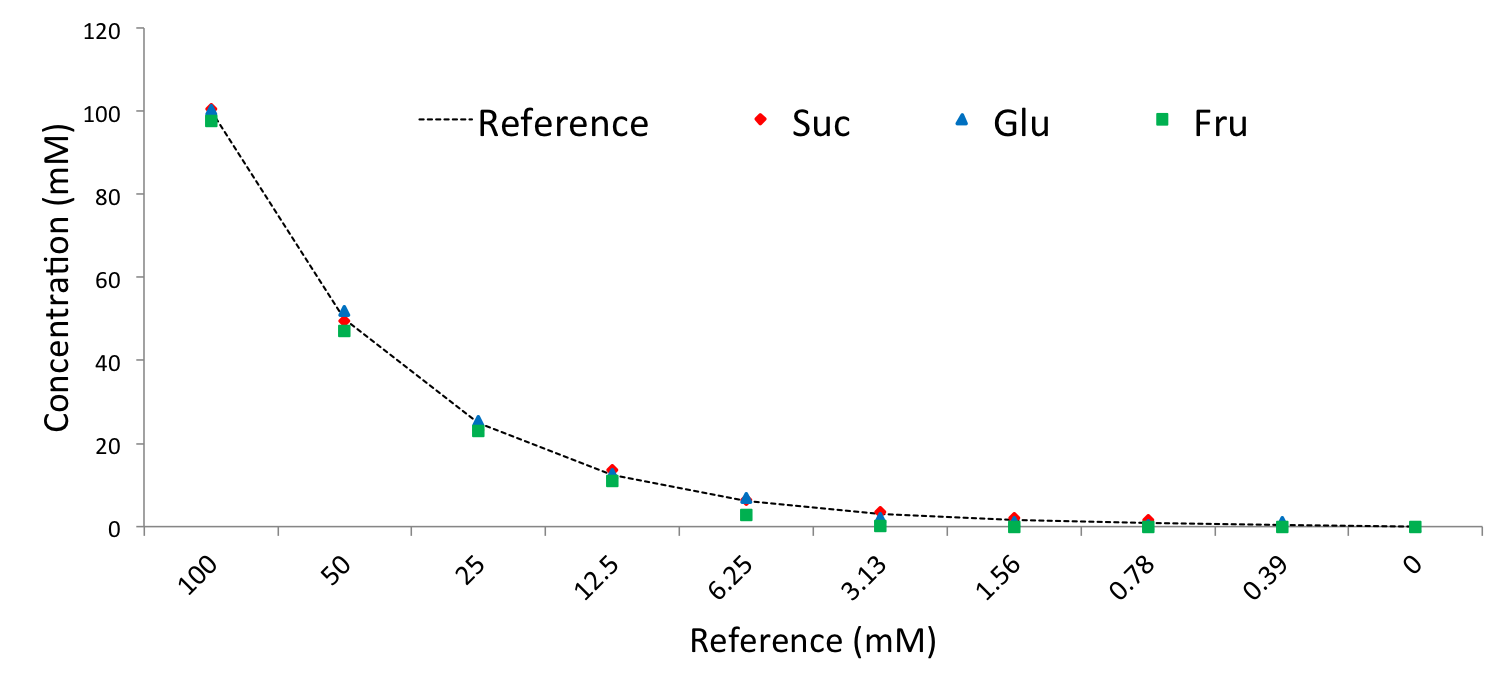


**Figure 1.** Limit of detection for the FTIR PLS predictive models for sucrose, glucose and fructose as determined by serial dilution of sugar mixtures.


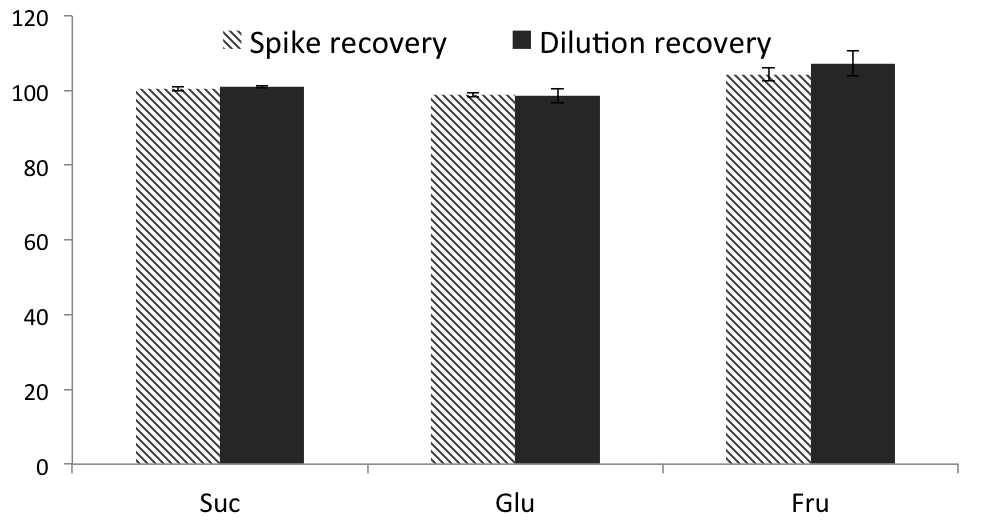


**Figure 2.** Three *Sorghum bicolor* juice samples were diluted and spiked with variable concentrations of sucrose, glucose, and fructose to determine the percentage recovery of each sugar using the FTIR PLS prediction models.
