# Supplementary figures and images for: A holistic high-throughput screening framework for biofuel feedstock assessment that characterises variations in soluble sugars and cell wall composition in Sorghum bicolor
Source: Biotechnol Biofuels. 2013 Dec 23;6:186. doi: 10.1186/1754-6834-6-186 (PMC3892131; doi:10.1186/1754-6834-6-186)

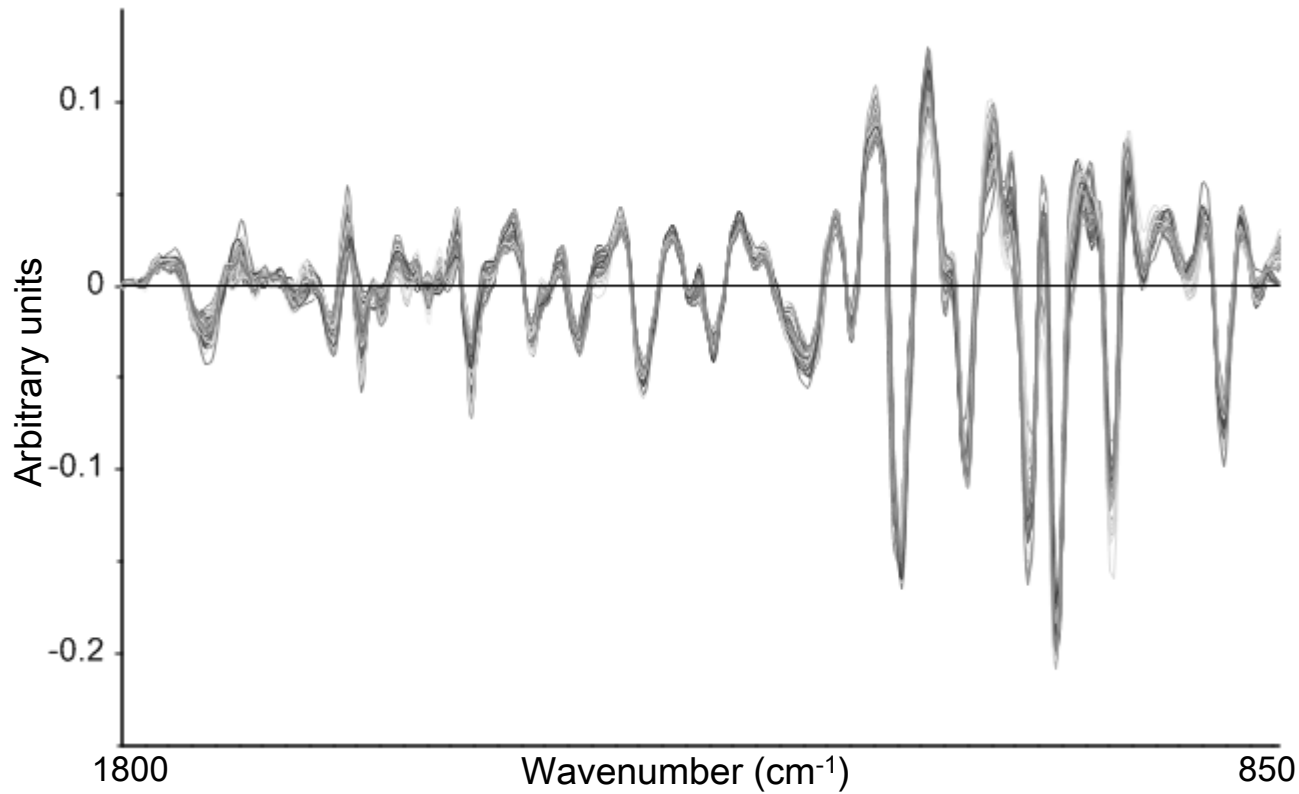

Supplement: Additional file 8 — Processed spectra used to calibrate the digestibility PLS model. Second derivative spectra with an EMSC applied, which were used to calibrate the digestibility PLS model. EMSC, extended multiplicative scatter correction; PLS, partial least squares. [file 1754-6834-6-186-S8.pdf]
