# Supplementary material for: A holistic high-throughput screening framework for biofuel feedstock assessment that characterises variations in soluble sugars and cell wall composition in Sorghum bicolor
Source: Biotechnol Biofuels. 2013 Dec 23;6:186. doi: 10.1186/1754-6834-6-186 (PMC3892131; doi:10.1186/1754-6834-6-186)
Supplement: Additional file 9 — PLS digestibility model diagnostics and band assignment chart. Model diagnostics for the PLS digestibility model showing clear separation of digestibility in the scores plot and a representation of cell wall peaks in the regression coefficients. A band assignment chart is displayed for reference. PLS, partial least squares. [file 1754-6834-6-186-S9.docx]

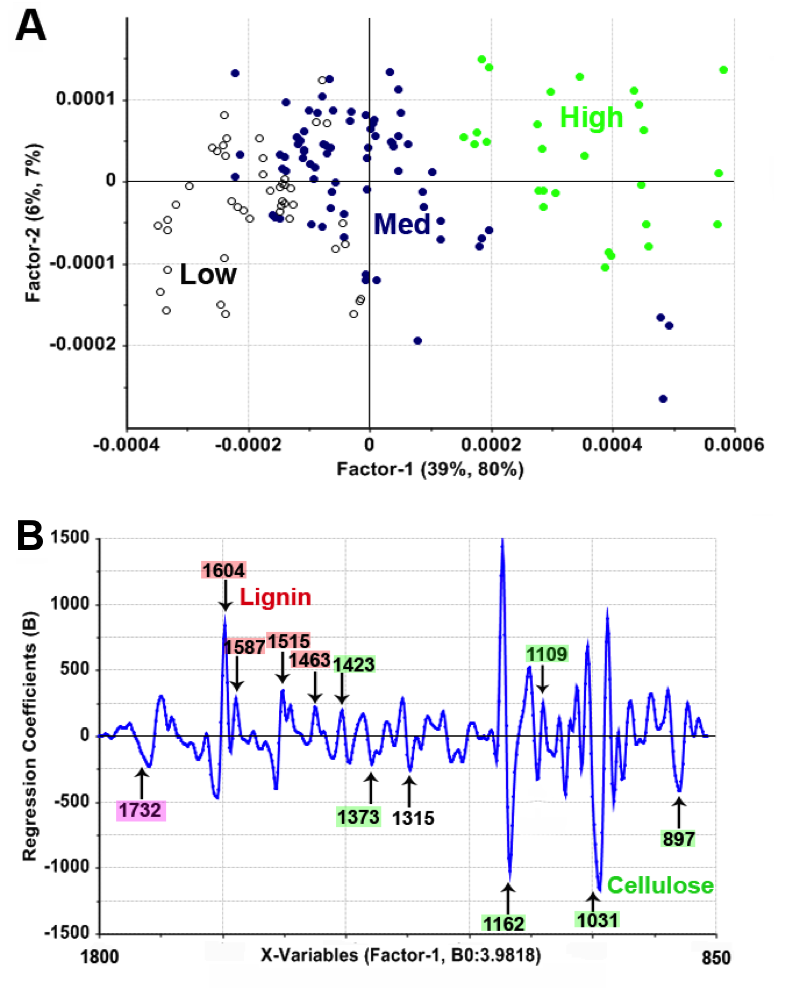


**Figure 1.** Digestibility PLS prediction model diagnostics showing **A)** Scores plot of LV1 against LV2 with samples colour coded as high (8-10.5 μg.mgDW^-1^.h^-1^; green), medium (4-8 μg.mgDW^-1^.h^-1^; blue), and low (0-4 μg.mgDW^-1^.h^-1^; black outline) **B)** LV1 regression coefficients plot showing the main peaks that contributed to LV1 (Units are arbitrary and peaks corresponds to the peak assignment table below (**table 1**) with red, green and purple representing peaks associated with lignin, cellulose and hemicellulose respectively).

**Table 1** FTIR peak assignment in the cell wall fingerprint region (1800-850 cm^-1^) identified by reference to the literature
