# Supplementary material for: A holistic high-throughput screening framework for biofuel feedstock assessment that characterises variations in soluble sugars and cell wall composition in Sorghum bicolor
Source: Biotechnol Biofuels. 2013 Dec 23;6:186. doi: 10.1186/1754-6834-6-186 (PMC3892131; doi:10.1186/1754-6834-6-186)
Supplement: Additional file 10 — Whole stalk fermentable sugar calculations. Whole stalk calculations for fermentable sugars in the soluble sugar fraction and cell wall fraction resulting in a total fermentable sugar yield calculation. [file 1754-6834-6-186-S10.docx]

**Whole stalk calculations**

**Whole stalk juice sugar**

$$Whole stalk solule sugar yield ({[TS]}_{WSY}) \left( g \right)= {[S]}_{adj}.V_{adj}$$

where $V_{adj}= \frac{\frac{(\frac{{FW}_{I}}{{FW}_{r}}- \frac{{DW}_{I}}{{DW}_{r}})}{1000}}{V_{ratio}}$

and ${[TS]}_{\boldsymbol{adj}}\boldsymbol{=}{MW}_{\boldsymbol{g\&f}}\boldsymbol{(}\frac{\left[ glucose \right]_{I}}{\left[ glucose \right]_{r}}+ \frac{\left[ fructose \right]_{I}}{\left[ fructose \right]_{r}})+ {MW}_{s}(\frac{\left[ sucrose \right]_{I}}{\left[ sucrose \right]_{r}})$

[*S*]*_adj_* = total sampled internode sugar (adjusted) (g/L)

*V_adj_* = 4^th^ internode juice volume (adjusted) (L)

*V_ratio_* = volumetric ratio (as a decimal i.e. 0.11)

*FW_I_* = fresh weight of the sampled internode (g)

*FW_r_* = fresh weight density ratio (experimentally determined **Figure 2**)

*DW_I_* = dry weight of the sampled internode (g)

*DW_r_* = dry weight density ratio (experimentally determined **Figure 2**)

*MW_g&f_* = molecular weight of glucose and fructose (180.16)

*MW_s_* = molecular weight of sucrose (342.3)

*[glucose]_I_* = glucose concentration of sampled internode (M)

*[glucose]_r_* = glucose concentration ratio (experimentally determined **Figure 2**)

*[fructose]_I_* = fructose concentration of sampled internode (M)

*[fructose]_r_* = fructose concentration ratio (experimentally determined **Figure 2**)

*[sucrose]_I_* = sucrose concentration of sampled internode (M)

*[sucrose]_r_* = sucrose concentration ratio (experimentally determined **Figure 2**)

**Whole stalk cell wall hydrolysis yield**

$$Whole stalk cell wall hydrolysis yield ({TD}_{WSY}) \left( g \right)= D_{adj}.B_{adj}$$

where $B_{adj}=\frac{(\frac{{DW}_{I}}{{DW}_{r}})}{V_{ratio}}$

and $D_{\boldsymbol{adj}}\boldsymbol{=} \frac{\boldsymbol{D}_{\boldsymbol{I}}}{\boldsymbol{D}_{\boldsymbol{r}}}$

*B_adj_ = biomass of the sampled internode (adjusted) (g)*

*D_adj_ = digestibility of the sampled internode (adjusted) (μg.mgDW^-1^.h^-1^)*

*D_I_ = digestibility of sampled internode (μg.mgDW^-1^.h^-1^)*

*D_r_ = digestibility rate ratio (determined experimentally* ***Figure 2****)*

**Total stalk fermentable sugar yield calculation**

*Whole stalk total fermentable sugar yield (g)*  $=\left[ TS \right]_{WSY}+ {TD}_{WSY}$

$={[S]}_{adj}.V_{adj}+D_{adj}.B_{adj}$

*(as presented in main text)*
